# Dysregulation of anti-Mullerian hormone expression levels in mural granulosa cells of *FMR1* premutation carriers

**DOI:** 10.1038/s41598-021-93489-x

**Published:** 2021-07-08

**Authors:** Moran Friedman-Gohas, Raoul Orvieto, Abigael Michaeli, Adva Aizer, Michal Kirshenbaum, Yoram Cohen

**Affiliations:** 1 Sackler Faculty of Medicine, Tel Aviv University, Tel Aviv, Israel; 2 Infertility & IVF Unit, Chaim Sheba Medical Center, Tel Hashomer, 5262000 Ramat Gan, Israel; 3 The Tarnesby-Tarnowski Chair for Family Planning and Fertility Regulation at the Sackler, Faculty of Medicine, Tel Aviv University, Tel Aviv, Israel

**Keywords:** Gene expression, Infertility

## Abstract

*FMR1* premutation (55–200 CGG repeats) results in fragile X-associated primary ovarian insufficiency (FXPOI). We evaluated expression levels of folliculogenesis-related mediators, follicle-stimulating hormone (FSH) receptor and anti-Mullerian hormone (AMH), to gain insights into the mechanisms underlying the reduced ovarian function. Mural granulosa cells (MGCs) were collected from *FMR1* premutation carriers and noncarriers undergoing IVF treatments. At baseline, MGCs of carriers demonstrated significantly higher mRNA expression levels of AMH (3.5 ± 2.2, n = 12 and 0.97 ± 0.5, n = 17, respectively; p = 0.0003) and FSH receptor (5.6 ± 2.8 and 2.7 ± 2.8, respectively; p = 0.02) and higher AMH protein expression on immunostaining. Accordingly, *FMR1* premutation-transfected COV434 cells exhibited higher AMH protein expression than COV434 cells transfected with 20 CGG repeats. We conclude that *FMR1* premutation may lead to dysregulation of AMH expression levels, probably due to a compensatory mechanism. Elucidating the pathophysiology of FXPOI may help in early detection of ovarian dysfunction and tailoring IVF treatments to *FMR1* premutation carriers.

## Introduction

Fragile X syndrome (FXS) is a trinucleotide repeat disorder commonly accompanied by male intellectual disability^1^. It is the major cause of inherited mental retardation^2^ and the prominent known reason for autism spectrum disorders^3^. Patients carrying the full CGG repeat expansion mutation (> 200 repeats) lack expression of the fragile X mental retardation 1 (*FMR1*) protein (FMRP) due to hypermethylation of the promoter region of the *FMR1* gene^4^. Carrying the *FMR1* premutation CGG repeat expansion (55–200 repeats) leads to two different phenotypes: fragile X-associated primary ovarian insufficiency (FXPOI) in females^5,6^ and fragile X-associated tremor/ataxia syndrome (FXTAS) mainly in males^7,8^.

Unlike individuals with the full mutation, female premutation carriers have elevated *FMR1* mRNA expression levels such that FMRP expression remains normal or only slightly decreased^4^. They have been shown to exhibit abnormal levels of ovarian reserve biomarkers, poor response to controlled ovarian hyperstimulation (COH), and early menopause^5,9^, all indicative of deteriorating fertility. Occurrences of primary ovarian insufficiency (POI) are mainly idiopathic. However, some etiologies such as autoimmune and infectious ovarian impairments, toxins and genetic aberrations have been associated with POI^10^. Within *FMR1* premutation carriers, about 20% have POI and diminished ovarian reserve compared to 1% of the general population^11^.

Several studies have reported an association of premature ovarian insufficiency with dysregulations of folliculogenesis-related genes, including genes coding stem cell factor (SCF)^12^, growth and differentiation factor 9 (GDF9)^13^, follicle-stimulating hormone (FSH), and anti-Mullerian hormone (AMH)^14,15^. AMH, secreted by granulosa cells, might be involved in the recruitment of dormant primordial follicles^12^. AMH's inhibitory role is suspected to assist in dominant follicle selection, possibly by reducing the follicle's sensitivity to FSH stimulation^16,17^. Moreover, it seems that the role of AMH during follicular development is stage dependent, and species specific^18^. Studies have suggested that AMH promotes pre-antral follicle growth, but inhibits antral follicle maturation and dominant follicle selection in rats, non-human-primates and humans^18,19^. We hypothesized that an imbalance in folliculogenesis mediators may be involved in the decreased fertility of female *FMR1* premutation carriers. The aim of the present study was to evaluate the mRNA and protein expression of AMH, a well-defined marker of ovarian reserve, and FSH receptor in mural granulosa cells (MGCs) of *FMR1* premutation carriers and noncarriers undergoing in vitro fertilization (IVF) treatments and in *FMR1* premutation-transfected tumor cells in order to gain insight into the pathophysiologic mechanisms underlying FXPOI.

## Results

**Figure 1:**
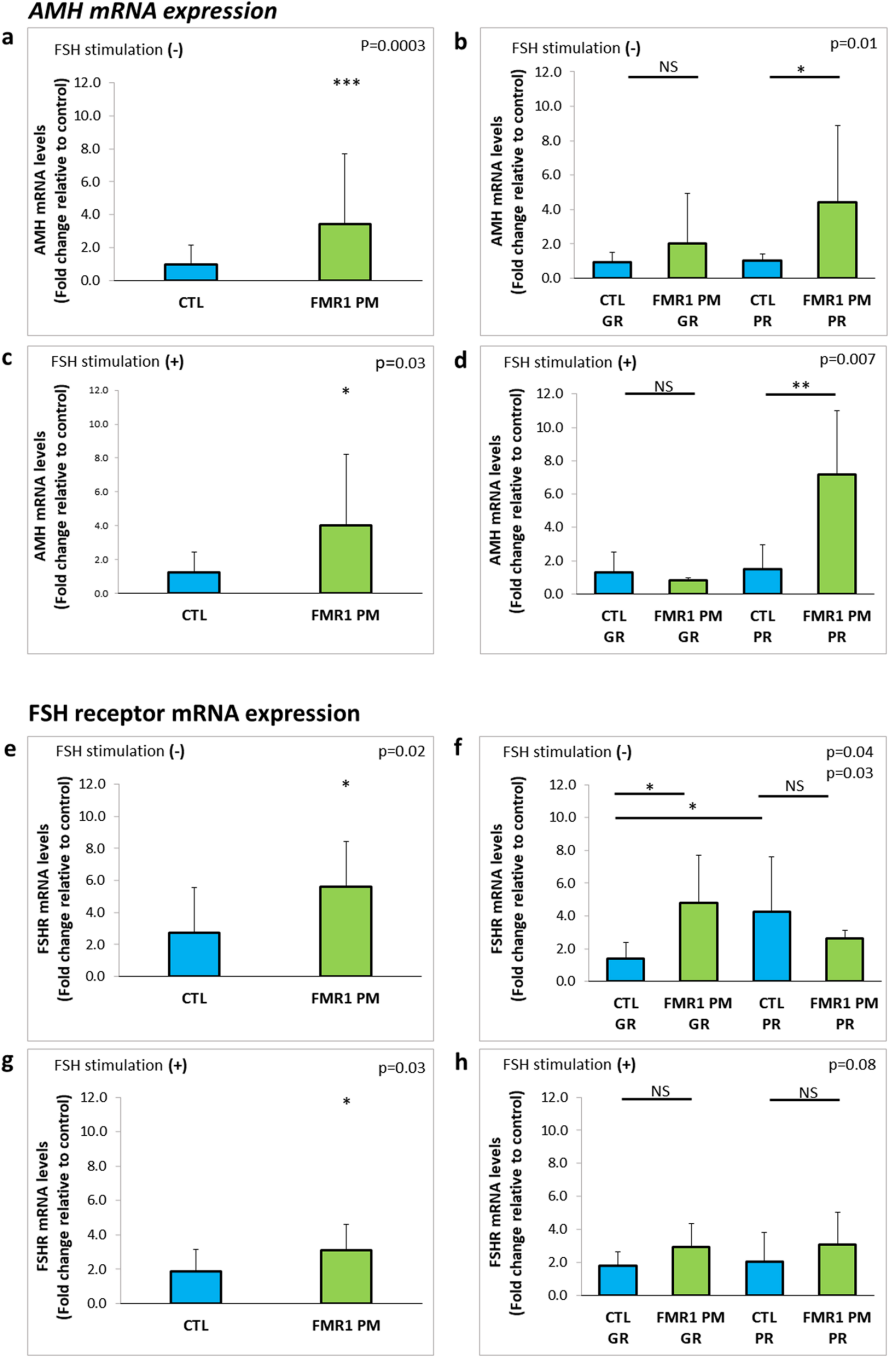
Higher AMH and FSH receptor expression levels in MGCs of *FMR1* premutation carriers compared to MGCs of noncarriers. (**a**) AMH and (**e**) FSH receptor mRNA expression levels in MGCs of *FMR1* premutation carriers (*FMR* PM) and non-carriers (CTL) without FSH stimulation. (**b**) AMH and (**f**) FSH receptor mRNA expression levels in MGCs in sub-groups according to the oocytes number retrieved in OPU; GR > 5, PR ≤ 5. (**c**) AMH and (**g**) FSH receptor mRNA expression levels in MGCs following 48 h of FSH stimulation. (**d**) AMH and (**h**) FSH receptor mRNA expression levels in FSH-stimulated MGCs by number of oocytes number retrieved in OPU: GR > 5, PR ≤ 5. *p < 0.05, **p < 0.01, ***p < 0.001. Graphs show fold change in expression relative to control ± STDEV.

Higher AMH and FSH receptor mRNA expression levels were found in *FMR1* premutation carriers. At baseline (before FSH stimulation), MGCs of *FMR1* premutation carriers demonstrated higher AMH mRNA expression levels than MGCs of noncarriers (3.5 ± 2.2, n = 12 and 0.97 ± 0.5, n = 17 respectively; p = 0.0003 (Fig. 1a). Stratification of the groups by response to COH (≤ 5 or > 5 oocytes produced) showed that MCGs of poor responders who carried the *FMR1* premutation had significantly higher AMH mRNA levels than MCGs of poor responders without the premutation (3.97 ± 2.7, n = 7 and 1.0 ± 0.4, n = 9, respectively; p = 0.01) (Fig. 1b). After 48 h of FSH stimulation, AMH expression was still higher (by 3.2 fold) in MCGs of the carriers than the noncarriers (p = 0.03) (Fig. 1c) and in MCGs of poor responders in the carrier group than poor responders in the noncarrier group (7.9 ± 3.8, n = 5 and 1.5 ± 1.4, n = 8 p = 0.007, respectively) (Fig. 1d).

Baseline expression levels of FSH receptor mRNA were significantly higher (by 2.1 fold) in *FMR1* premutation carriers than noncarriers (p = 0.02) (Fig. 1e). Stratification of the groups by response to COH showed a significantly higher FSH receptor expression level (by 3.4 fold) in MCGs of good responders in the carrier group than in MCGs of good responders in the noncarrier group (p = 0.04, n = 7 and n = 9 respectively). However, there was a nonsignificant trend-level decrease in FSH receptor expression in MCGs of poor responders in the carriers group compared to the noncarriers group (n = 5) (Fig. 1f). Indeed, within the noncarrier group, poor responders had a higher FSH receptor mRNA level (by 3.1fold) than good responders (p = 0.03; n = 6 and n = 9 respectively) (Fig. 1f). After FSH stimulation, premutation carriers had significantly higher base line levels of FSH receptor mRNA expression than noncarriers (3.1 ± 1.5, n = 12 and 1.9 ± 1.2, n = 14, respectively; p = 0.03) (Fig. 1g). However, FSH stimulation did not instigates significant differences between the groups (Fig. 1h).

**Figure 2:**
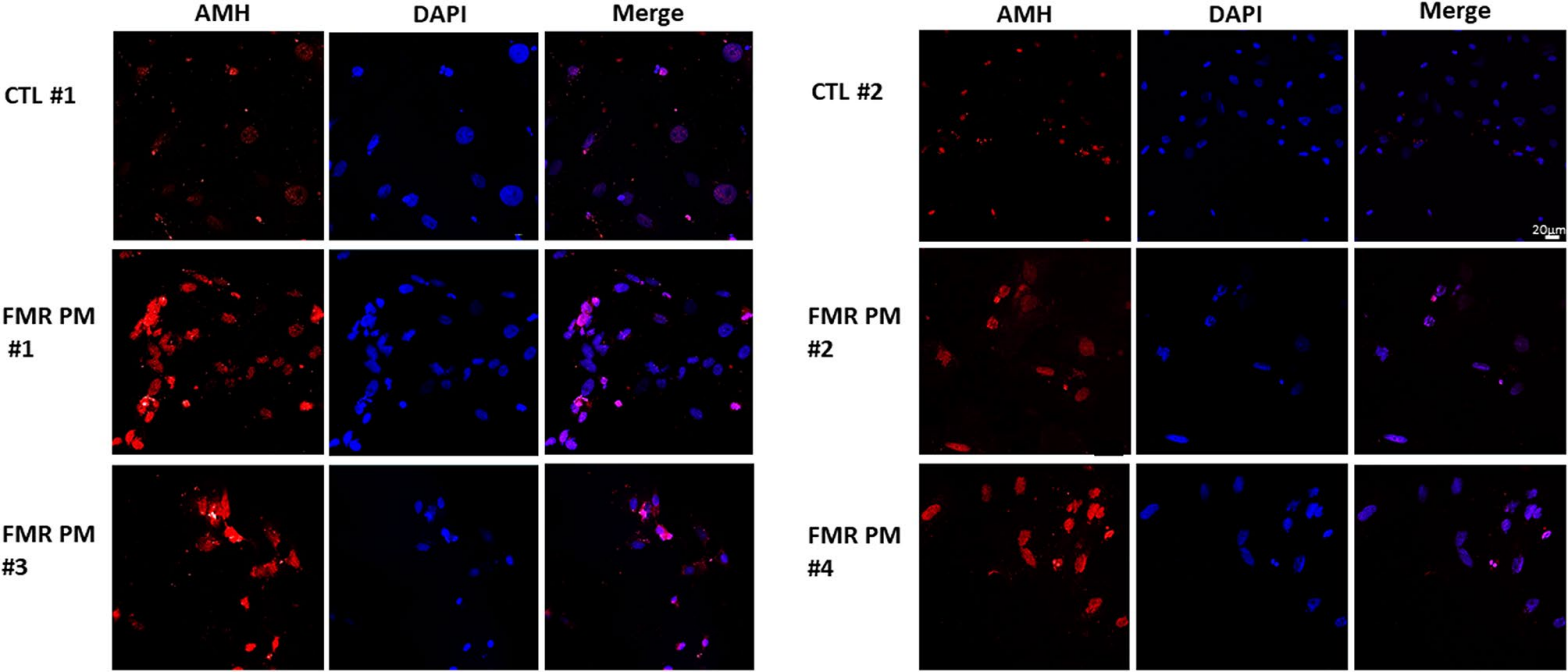
Higher AMH protein expression in MCGs of *FMR1* premutation carriers compared to MGCs of noncarriers. Fixated and stained MGCs from *FMR1* premutation carriers (*FMR* PM #1–4) displaying higher AMH expression compared to noncarriers (CTL #1–2). Bar = 20 µm.

Following detection of a dysregulation in mRNA expression of folliculogenesis-related genes in MGCs from *FMR1* premutation carriers, we explored AMH protein expression. Immunostaining with anti-AMH antibody revealed an elevated protein expression in MGCs of *FMR1* premutation carriers compared to MGCs of noncarriers (Fig. 2).

**Figure 3:**
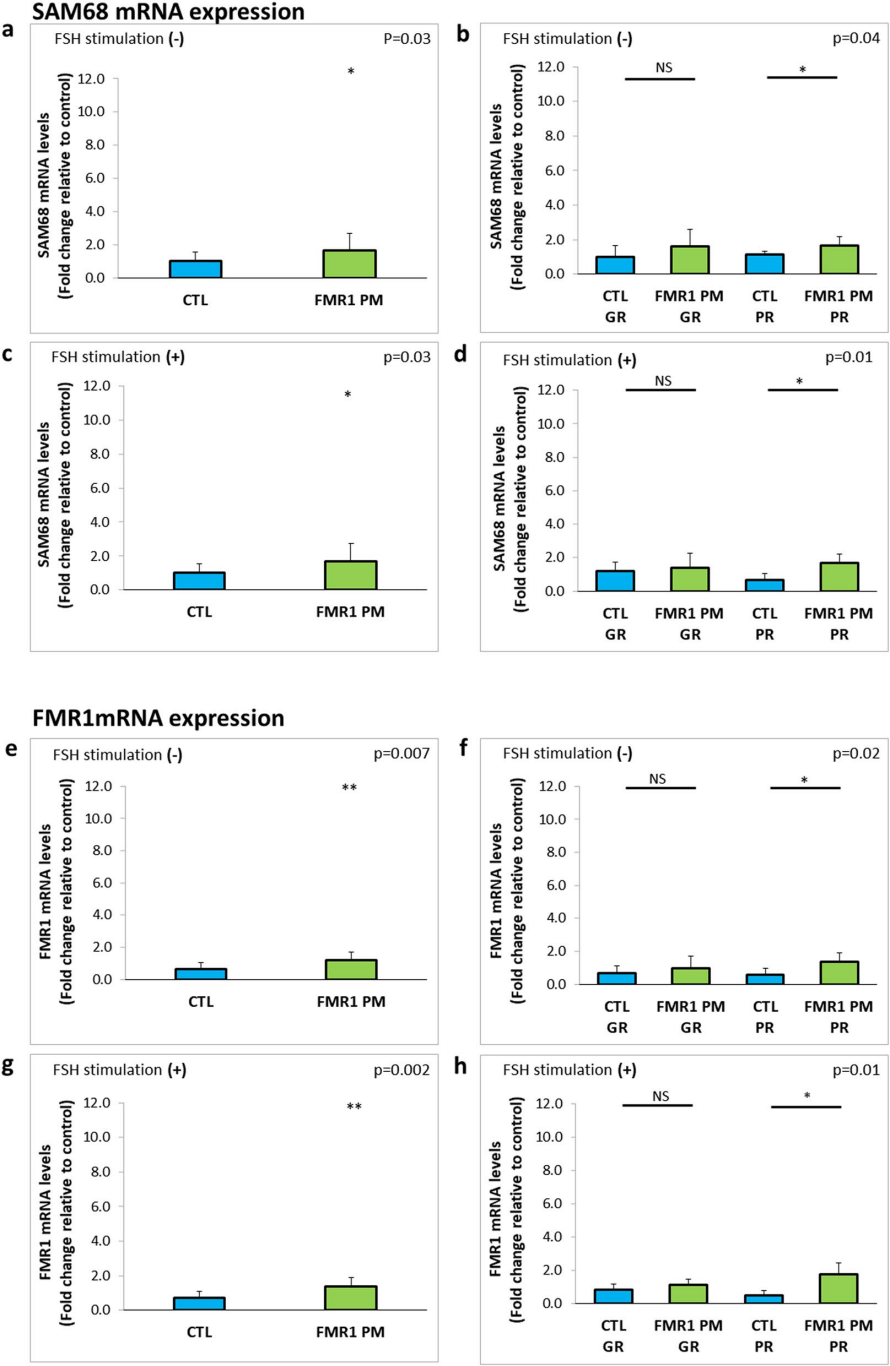
Higher SAM68 and *FMR1* mRNA expression levels in MGCs of *FMR1* premutation carriers compared to noncarriers. (**a**) SAM68 and (**e**) *FMR1* mRNA expression levels in MGCs of *FMR1* premutation carriers (*FMR* PM) and noncarriers (CTL) without FSH stimulation. (**b**) SAM68 and (**f**) *FMR1* mRNA expression levels in MGCs in sub-groups according to the oocytes number retrieved in OPU; GR > 5, PR ≤ 5. (**c**) SAM68 and (**g**) *FMR1* mRNA expression levels in MGCs following 48 h of FSH stimulation. (**d**) SAM68 and (**h**) *FMR1* mRNA expression levels in FSH-stimulated MGCs by number of oocytes retrieved in OPU: GR > 5, PR ≤ 5. *p < 0.05, **p < 0.01, ***p < 0.001. Graphs show fold change relative to control expression ± STDEV.

Src-associated substrate in mitosis of 68 kDa (SAM68) is a RNA-binding protein that plays an important role in RNA signal transduction. Evaluation of baseline expression of SAM68 mRNA revealed significantly higher levels in MGCs of *FMR1* premutation carriers than noncarriers (1.6 ± 0.8, n = 12 and 1.0 ± 0.5, n = 17, p = 0.03 respectively). The same trend was observed following FSH stimulation (1.7 ± 1.0, n = 12 and 1.0 ± 0.5, n = 17, p = 0.03 respectively) (Fig. 3a,c). On comparison of poor responders between the groups, expression levels of *SMA68* were significantly higher in carriers than noncarriers both before and after FSH stimulation (Fig. 3b,d).

*FMR1* mRNA expression levels were also higher in premutation carriers than non-carriers (1.2 ± 0.5, n = 11 and 0.6 ± 0.4, n = 14, respectively; p = 0.007) (Fig. 3e). A twofold increase was observed following FSH stimulation (p = 0.002) (Fig. 3g). Similar findings were noted on analysis of poor responders both before and after FSH stimulation (Fig. 3f,h).

**Figure 4:**
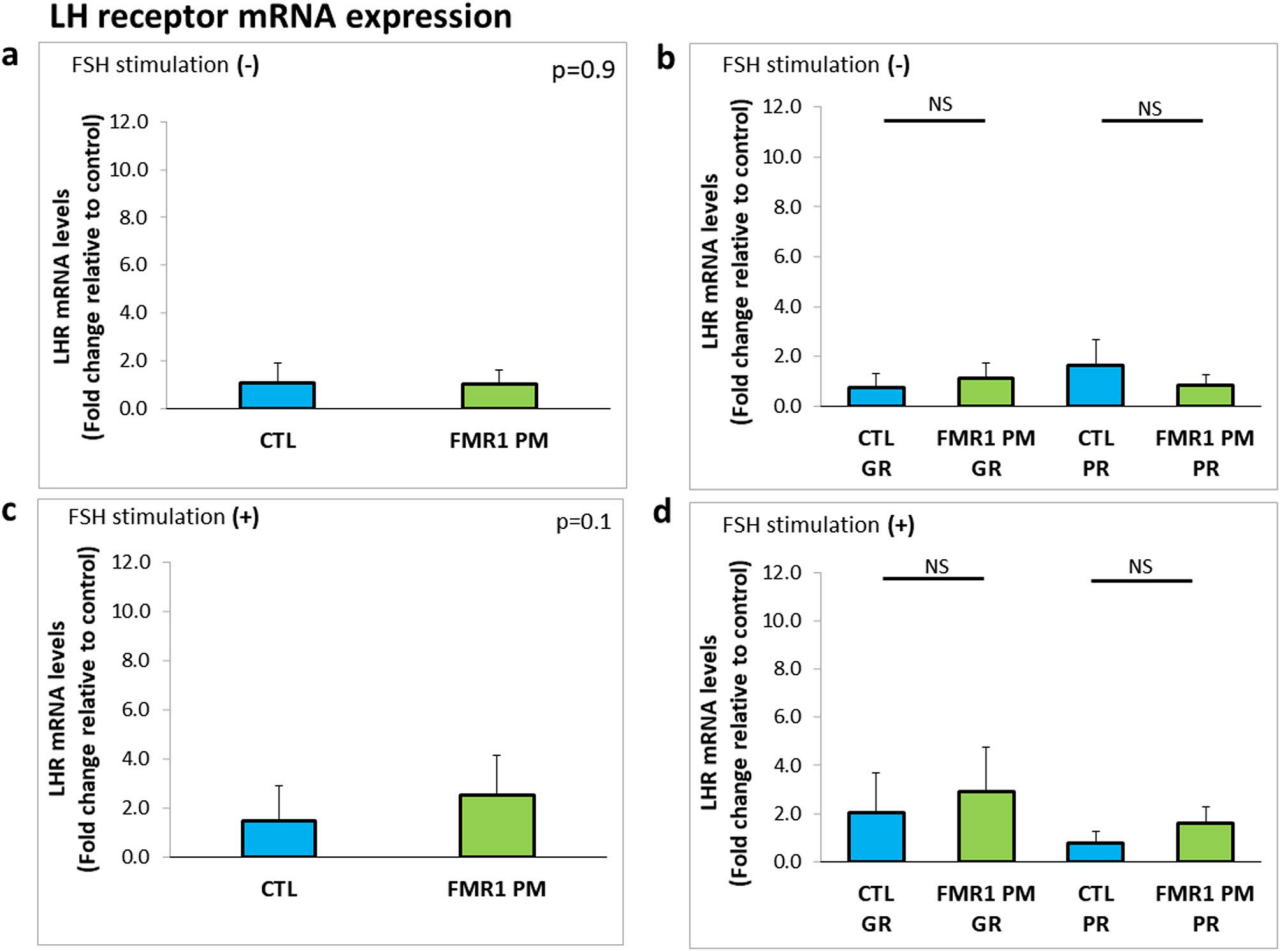
Similar LHR mRNA expression levels in MGCs of *FMR1* premutation carriers and noncarriers. (**a**) Expression levels in MGCs of *FMR1* premutation carriers (*FMR* PM) and non-carriers (CTL) without FSH stimulation. (**b**) Expression levels in MGCs by number of oocytes retrieved in OPU: GR > 5, PR ≤ 5. (**c**) Expression levels in MGCs following 48 h of FSH stimulation. (**d**) Expression levels in FSH-stimulated MGCs by number of oocytes retrieved in OPU: GR > 5, PR ≤ 5. Graphs show fold change relative to control expression ± STDEV.

We cultured the human isolated MGCs (obtained following follicular puncture at the end of an ovarian stimulation) for 4 days in FSH-deprived conditions to achieve a steady state resembling antral preovulatory follicles (described in the methods). To confirm that, we examined the expression levels of luteinizing hormone (LH) receptor in MGCs from *FMR1* premutation carriers and noncarriers. No between- or within-group differences in expression were noted (Fig. 4a–d).

**Figure 5:**
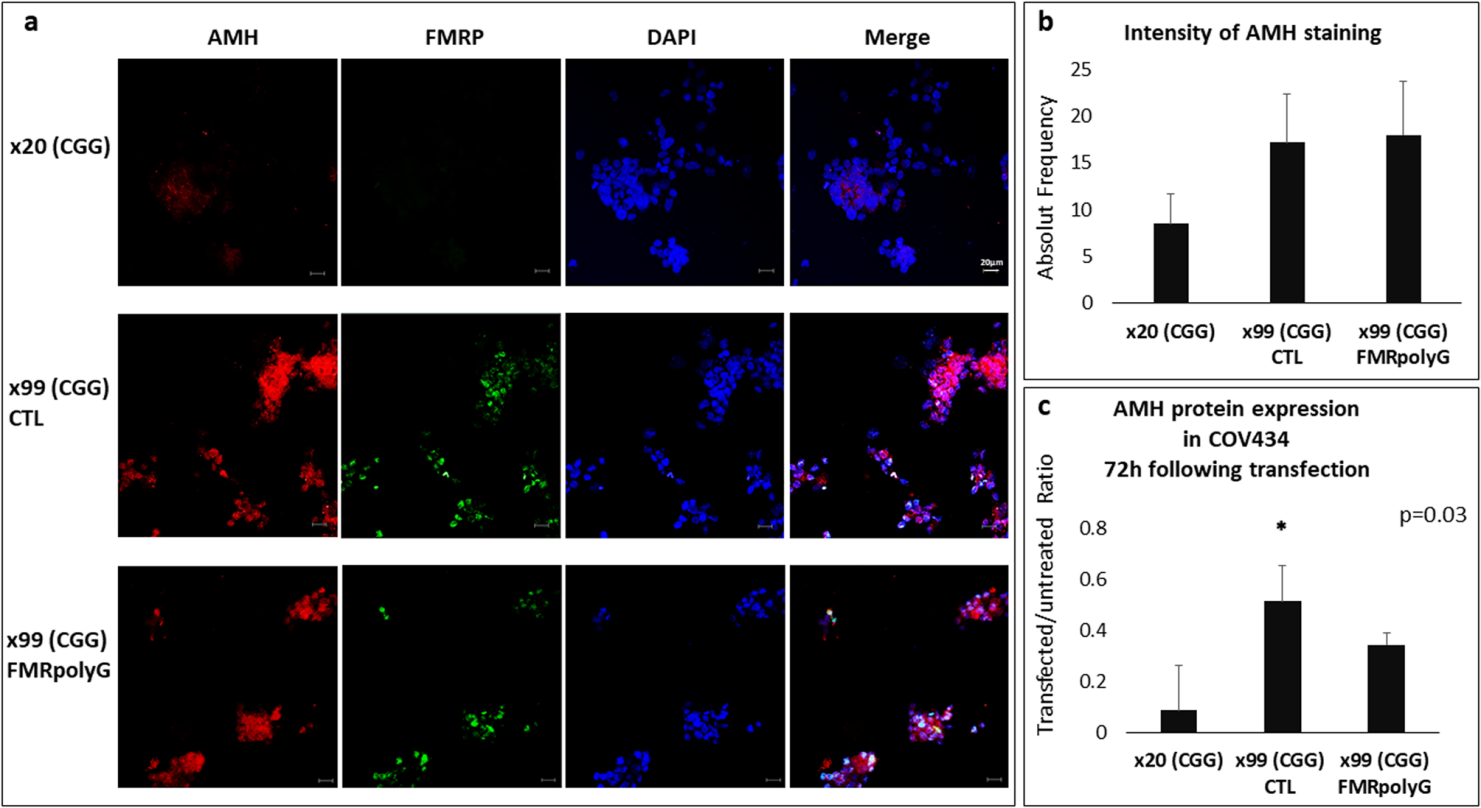
COV434 premutation model expressing higher AMH once in the premutation range. (**a**) 99xCGG-transfected COV434 cells displaying higher AMH expression than 20xCGG- transfected COV434 cells. GFP signal represents transfected cells. Bar = 10 µm. (**b**) Quantification of AMH expression in transfected COV434 cells shown in panel (**a**). (**c**) Western blot showing higher expression in COV434 transfected with 99xCGG with and without FMRpolyG expression. Values were normalized with untreated COV434. All groups were harvested 72 h following transfection.

Given the dysregulation of AMH expression in the *FMR1* premutation carriers, we investigated the impact of CGG repeats on AMH expression. We examined AMH expression in COV434 cells transfected with 99 CGG repeats, with and without FMRpolyG expression (GFP signal represented transfected cells) compared to COV434 cells transfected with 20 CGG repeats. Following immunostaining with AMH antibody, we noted higher AMH protein expression in the premutation model of transfected COV434 cells regardless of the ability of the cells to produce the toxic FMRpolyG protein (Fig. 5).

The clinical and IVF cycle characteristics of the *FMR1* premutation carriers and noncarriers are shown in Table 1.

**Table 1:** Characteristics of *FMR*1 premutation carriers and noncarriers.

Characteristics	FMRI permutation noncarriers (n = 17)	FMR1 premutation carriers (n = 12)	p value
Age (years)	34.2 ± 9.6	33.4 ± 4.6	0.69
Parity	0.8 ± 1.2	1.3 ± 1.5	0.24
Basal FSH (IU/L)	6.7 ± 2.9	7 ± 2.0	0.76
Basal LH (IU/L)	4.7 ± 2.1	4.3 ± 1.4	0.62
Basal FSH/LH ratio	1.4	1.6	
Total gonadotrophins used for stimulation (IU)	2946.2 ± 1469.6	3596.8 ± 1540.0	0.29
Peak estradiol (pmol/L)	5403.2 ± 2355.4	5336.7 ± 2963.4	0.95
No. of oocytes retrieved	9.6 ± 5.3	9.5 ± 6.5	0.98
No. of pregnancies	0.9 ± 1.4	0.3 ± 0.5	0.58
FMR1 repeats (range)	< 55	64–199	

Values given as mean and standard deviation unless otherwise indicated.

*FSH* follicle-stimulating hormone, *LH* luteinizing hormone.

## Discussion

In the present study, we observed elevated levels of AMH and FSH receptor at both the RNA and protein levels in *FMR1* premutation carriers, suggesting a dysregulation in ovarian folliculogenesis.

The mechanism underlying the pathogenesis of FXPOI syndrome is poorly understood. We hypothesized that dysregulations in mediators of folliculogenesis were involved in the decline in fertility of *FMR1* premutation carriers. Our analysis of the expression of FSH receptor and AMH, major participants in follicular recruitment, growth, and maturation^14,15,20^, revealed higher levels in carriers of the *FMR1* premutation than in noncarriers. In light of the high diversity in expression within the groups at the mRNA (Fig. 1a–d) and protein (Fig. 2) levels, we further stratified them by response to COH. (We abstained stratifying according to CGG repeats number' due to the small sample size of only three *FMR1* premutation carriers, which is not statistically powered). We found that carriers who were poor responders (i.e., produced < 5 oocytes in response to COH for IVF) had an elevated AMH expression (Fig. 1b).

AMH is presumed to play many key roles in folliculogenesis. Although levels in serum remain constant during the menstrual cycle, the expression pattern of AMH in granulosa cells differ. In previous studies, AMH expression was first detected in granulosa cells of early primary follicles and was highest in preantral and antral follicles^14,21^. Using a rhesus macaque model, Xu et al*.* demonstrated that the activity of AMH in the ovary of primates could be stage-dependent, encouraging preantral follicle growth. AMH is associated with the recruitment of dormant follicles at the beginning of the cycle and with inhibiting further maturation of antral follicles in the late follicular stage, as part of the negative feedback process to assist selection of the dominant follicle^20^. Kedem et al*.* found that in women with polycystic ovarian syndrome (PCOS), there was a negative correlation between the size of antral follicles and expression levels of AMH in granulosa cells. Note that in PCOS the higher AMH expression is due to the high number of small follicles secreting AMH, reflecting the intrinsic characteristics of their granulosa cells. However, among patients undergoing in vitro maturation, levels were higher in older women with lower ovarian reserve than in younger ones^22^, supporting a compensatory role of AMH in promoting pre-antral follicle growth and increasing the antral-follicle pool. In another study by Kedem et al.^23^, higher AMH mRNA expression was detected in cumulus granulosa cells of large preovulatory follicles in non- metaphase II (MII) oocytes compared to MII oocytes. As with poor responder *FMR1* premutation carriers, abnormal follicullogenesis, manifested by either reduce oocytes cohort or abnormal maturation, might be the culprit of higher AMH mRNA expression, aiming to encourage preantral follicle growth. The role of AMH in folliculogenesis in humans has not been investigated in detail. It is generally presumed that AMH serves as a gate keeper, inhibiting primordial follicles recruitment (directly and via inhibiting FSH induced recruitment), mainly in rodents. Nevertheless, an additional possible mechanism might be related to an accelerated activation of dormant/primordial follicles by the higher expression of AMH in human *FMR1* premutation carriers. Further studies with a larger sample size, enabling the observation of earlier stages of primordial follicles recruitment, are required to determine and elucidate which mechanisms are involved in *FMR1* premutation carriers' pathophysiology.

*FMR1* premutation carriers are at higher risk of diminished ovarian reserve and in most cases demonstrate poorer response to COH. In accordance with the study of Kedem et al.^22^, we demonstrated significantly higher AMH expression levels in poor responders with a low oocyte yield. We therefore suggest that in *FMR1* premutation carriers, the early impaired follicle activity is followed by a compensatory mechanism that upregulates genes involved in folliculogenesis. In poor responders, the elevated expression of AMH and FSH receptor might initially accelerate follicular recruitment, but later it evokes a vicious cycle causing secondary damage to the ovarian reserve and resulting in POI. Moreover, the possibility of accelerating follicular recruitment in *FMR1* premutation carriers might explain our observations; women who carry the premutation exhibited higher AMH secretion, this might cause an upraised recruitment of dormant follicles in each menstrual cycle, and results in a decreased ovarian reserve. Some *FMR1* premutation carriers undergoing IVF treatments might obtain higher oocytes number following oocytes retrieval in the beginning, and less in later treatments. We found that the intensity of the AMH staining varied within the *FMR1* premutation group. Apparently in some *FMR1* premutation carriers another compensation mechanism might be involved and prevent them from deteriorating into a state of FXPOI. Further investigations are needed to understand the association between folliculogenesis mediators and to determine whether their elevation is the culprit or an epiphenomenon of POI.

Similar to poor responders, good responders in the *FMR1* premutation carrier group expressed significantly higher FSH receptor than noncarriers (Fig. 1f). Again, FSH receptor upregulation may be due to a compensatory process, mitigating a lower response of carrier follicles to COH. The trend of decreased expression levels in the poor responders implies that the compensatory mechanism is not functional, which would explain the decreased number of follicles retrieved from poor responders in the *FMR1* premutation carrier group. In good responders, however, FSH receptor levels are apparently elevated more efficiently, as suggested by the higher number of oocytes produced compared to poor responders. Within the noncarrier group, poor responders exhibited higher FSH receptor levels compared to good responders. Since FSH receptor has a role in the growth and maturation of the follicles, we suggest that when a lower number of oocytes are retrieved, an upregulation of FSH receptor expression might be evoked. While noncarriers' MGCs were able to elevate FSH receptor levels, *FMR1* premutation carriers' MGCs kept low levels of FSH receptor. To conclude this hypothesis, further investigation is needed with a larger cohort and data analysis comparing clinical characterizations.

The molecular mechanisms underlying FXTAS have been relatively explored and defined. Two major mechanisms were identified: RNA gain-of-function toxicity and toxic cryptic polyglycine-containing protein (FMRpolyG) expression (Sellier et al.^24,25^). Their role in the decline in fertility in individuals with the *FMR1* premutation is not known.

The RNA gain-of-function mechanism induces the CGG extended repeats tract to avidly sequester more than 30 RNA binding proteins, such as hnRNP A2, Pur α, SAM68, and Drosha and its partner DGCR8, thereby inducing their loss of function^8,24–28^). Sam68 has several functions in RNA editing one of which is alternative splicing. High expression levels of Sam68 have been shown in the gonads. Bianchi et al.^29^ observed direct binding of Sam68 to FSH receptor mRNA causing its downregulation in ovaries of adult knockout females. Accordingly, we observed elevated Sam68 mRNA levels in MGCs of *FMR1* premutation carriers (Fig. 3a–d) both before and after FSH stimulation. Interestingly, when the groups were stratified by response, the poor responders in the carrier group maintained the significantly high Sam68 expression levels relative to poor responders in the noncarrier group. Immunostaining with Sam68 antibody did not distinguish *FMR1* premutation MGCs from normal CGG repeat tract cells (Supplementary Fig. 1). Nevertheless, the elevation in mRNA levels might imply impaired Sam68 function which could trigger an upregulation of FSH receptor to compensate for the loss of function. These interactions between Sam68 and FSH receptor might play a role in FSH receptor dysregulation in *FMR1* premutation carriers and warrant further investigation.

*FMR1* mRNA levels were elevated in the *FMR1* premutation group (Fig. 3e–h), in accordance with previous reports^30,31^. Stratifying the premutation carrier group to poor and good responders, revealed that the elevation in *FMR1* expression levels was significant in the poor responder group only. For further conclusions regarding the relation between *FMR1* expression levels and the ovarian response of the carriers, a larger sample size is required. Together the increased levels of Sam68 and of *FMR1* suggests that RNA toxicity might be involved in FXPOI pathobiology.

Todd et al.^32^ proved that the CGG expansion prompts non-AUG-initiated (RAN) translation of mainly FMRpolyG, which accumulates in a large ubiquitin-positive inclusion body in the nervous system, and Buijsen et al.^33^ reported inclusions in ovarian stromal cells, but not in follicles of female *FMR1* premutation carriers. In a recent study in our laboratory evaluating the suitability of premutation-transfected COV434 cells as a disease model, we noted similar FMRpolyG expression to MGCs from *FMR1* premutation carriers^34^. In the present study, we used the transfected COV434 disease models (99 CGG repeats with or without the ability to express the FMRpolyG protein) to investigate the expression of AMH. Immunostaining and western blot revealed significant elevations of AMH elevations regardless of FMRpolyG expression (Fig. 5a–c). Therefore, we postulate that the premutation state (55–200 CGG repeats) might be a primary cause of the overexpression of AMH due to a compensatory mechanism via upregulation of folliculogenesis mediators, and FMRpolyG might not be associated with the dysregulation in AMH expression.

In conclusion, we demonstrated a dysregulation of the folliculogenesis regulators, FSH receptor and AMH. The association between the elevated levels of these regulators and the proposed RNA and protein toxicity mechanisms in FXPOI remains unclear. The present study is an important step in elucidating the consequences of the *FMR1* premutation range and should facilitate future studies of the pathophysiology of FXPOI. The findings may have implications for improving the early detection of deteriorating fertility and developing IVF treatments tailored to *FMR1* premutation carriers.

## Material and methods

### Experimental model and subject details

Mural granulosa cells (MGCs) of women undergoing IVF treatments.

Ethics approval was granted by the Institutional Ethics Review Board of Sheba Medical Center, Israel (8707-11-SMC and 6140-19-SMC), and all patients provided written informed consent. In Israel, all women are encouraged to undergo genetic testing before conceiving, including *FMR1* CGG carrier status. Therefore, the CGG repeat number is known for all *FMR1* premutation carriers (55–200 CGG repeats) and noncarriers (< 55 CGG repeats). *FMR1* premutation carriers are often referred for IVF with pre-implantation genetic testing (PGT) to exclude embryos with pathological CGG expansion.

The study group included 12 *FMR1* premutation carriers referred for IVF-PGT who reached the ovum pick-up (OPU) stage. For the control group, the study patients were age-matched with 17 noncarriers undergoing IVF-intracytoplasmic sperm injection (ICSI) for male infertility during the same period at the same hospital. The controlled ovarian hyperstimulation (COH) protocol was determined by the treating physician. Variable doses of gonadotropins were administrated according to the woman's age and ovarian response in the previous treatment cycle. Doses were further adjusted on the basis of serum estradiol (E_2_) levels and follicle diameters measured by vaginal ultrasound every 2 or 3 days. Oocyte aspiration was performed under ultrasound guidance by the transvaginal route 36–38 h after human chorionic gonadotropin (HCG) injection. Mural granulosa cells (MGCs) were collected from pooled follicular fluids of each woman in both groups. The basic clinical and IVF treatment cycle characteristics are shown in Table 1.

### COV434 cell line for transfection

The human ovarian granulosa tumor cell line COV434 was purchased from Sigma-Aldrich (cat. No. ECACC 07071909; St. Louis, MO, USA) maintained according to the manufacturer’s instructions.

### Mural granulosa cell culture

MGC culture was performed as previously described by our group^33,34^. MGCs from follicular fluid were washed and plated on plastic tissue culture plates containing Dulbecco’s Minimum Essential Medium (DMEM) with 5% fetal bovine serum, 1% l-Glutamine, and 1% penicillin/streptomycin, all from Biological Industries (Beit Haemek, Israel). Starting at 24 h after seeding, the culture medium was replaced every 24 h for 4 days. Half the cells from each *FMR1* premutation carrier were subjected to an additional 48 h of stimulation with 75 U/ml FSH. Cells were incubated at 37 °C in a humidified atmosphere of 5% CO_2_ in air.

### Transfection

Transfection was carried out as previously described^33^. COV434 cells were transfected with three plasmids generously provided by Dr. Charlet-Berguerand at the Department of Neurobiology and Genetics, University of Strasbourg, Illkirch, France^25,31,32^. In brief, two of the plasmids contained 99 CGG repeats, one expressing FMRpolyG and one not, in both plasmids the FMRP ORF is GFP-tagged, and the third (control) plasmid contained 20 CGG repeats (Without GFP tag). For the transfection, we used a mixture of 0.5 µg DNA (NucleoBond Xtra Midi Plus Kit, Macherey–Nagel, Duren, Germany), extracted according to the manufacturer's protocol, and 1.5 µl Mirus (Mirus Bio, Madison, WI, USA) at a final volume of 50 µl DMEM (Biological Industries).

### RNA isolation and molecular analysis

Total RNA was extracted using TRIzol reagent (Life Technologies, Invitrogen, Rhenium, Modi'in, Israel) followed by cDNA reverse transcription using the qScript cDNA Synthesis Kit (Quanta BioSciences, Beverly, MA, USA).

For real-time qualitative polymerase chain reaction (qPCR) analysis, we used the StepOnePlus System (Applied Biosystems, Waltham, MA, USA) with SYBR Green Fast assay (Invitrogen, Rhenium) under the following cycling parameters: 1 cycle at 95 °C for 20 s, 40 cycles each at 95 °C for 3 s and 60 °C for 30 s. Human beta-actin, a housekeeping gene, served as the control. Transcript expression levels were obtained by the relative quantification (DDCt) method using SDS software (Applied Biosystems).

Primer sequences were as follows:

- h-AMH_F: GCTGCCTTGCCCTCTCTAC.
- h-AMH_R: GAACCTCAGCGAGGGTGTT.
- h-FSHR_F: GAGAGCAAGGTGACAGAGATTCC.
- h-FSHR_R: CCTTTTGGAGAGAATGAATCTT.
- h-FMR1_F: AACAAAGGACAGCATCGCTAATG.
- h-FMR1_R: CAA ACGCAACTGGTCTACTTCCT.
- h-SAM68_F: GACTATGGACATGGGGAGGTTC.
- h-SAM68_R: ATTCCAGTCGTCCTGGCCAT.
- h-LHR_F: AGAGTGAACTGAGTGGCTGG.
- h-LHR_R: CAACACGGCAATGAGAGTAG.
- h-b-ACTIN_F: CCTGGACTTCGAGCAAGAGA.
- h-b-ACTIN_R: CAGCGGAACCGCTCATTGCCAATGG.

### Cell fixation and immunofluorescence

The permeabilization protocol consisted of 0.5% Triton X-100 in 4% paraformaldehyde (PFA) for 15 min, followed by incubation in 4% PFA for 20 min to fixate the cells; 5% bovine serum albumin (BSA) blocking solution was used to prevent nonspecific binding. Cells were incubated with the primary AMH antibody (Ab229212, 1:400, Abcam, Cambridge, UK) overnight at 4 °C followed by the secondary antibody (goat anti-rabbit IgG, Alexa Fluor 568, Abcam Ab175471, 1:200 RRID:AB_2576207) for 1 h at room temperature. Nuclear counterstaining was performed using DAPI Fluoromount-G (Southern Biotech, Birmingham, AL, USA). Images were acquired with a confocal microscope (LSM710, Zeiss, Oberkochen, Germany). The identical acquisition parameters were defined for both groups.

### Western blot analysis

Transfected COV434 cells were harvested and homogenized with a RIPA buffer, pH 7.4 (BioBasic, Toronto, Canada) containing phosphatase and protease inhibitors (Roche, Basel, Switzerland). A Bicinchoninic Acid Protein Assay Kit (Thermo Scientific, Rockford, IL, USA) was to calculate protein concentrations, and plates were read in a Varioskan multimode plate reader (Thermo Scientific). Criterion XT precast gels (4–20% bis–tris) (Biorad, Hercules, CA, USA) were loaded with 50 µg of total protein per well and transferred to a nitrocellulose membrane. Primary antibodies were anti-AMH (Ab229212, 1:2000, Abcam) and anti-glyceraldehyde 3-phosphate dehydrogenase (GAPDH) (Ab8245; 1:10,000, mouse monoclonal, RRID: AB2107448, Abcam). ChemiDocTM XRS + imager was used to scan the membrane, and Image Lab software (Biorad) assisted in quantifying the intensity of the bands of interest.

### Statistical analysis

Parameters were compared between groups using two-tailed unpaired Student's t-test. P values < 0.05 were considered statistically significant. (P values < 0.05 were marked*; < 0.01**; < 0.001***).

All methods were carried out in accordance with relevant guidelines and regulations.

## Supplementary Information

Supplementary Legends.

Supplementary Figure.

Supplementary Table.

## Data Availability

All reagents used in this study are available from the Lead Contact without restriction.

## Code availability

This study did not generate any unique datasets or code.

## Data availability

All data generated or analyzed during this study are included in this published article or in the data repositories listed in References. Upon request further data will be provided by the Lead Contact, Yoram Cohen (ycohen1@gmail.com).
