# Supplementary material for: Dysregulation of anti-Mullerian hormone expression levels in mural granulosa cells of FMR1 premutation carriers
Source: Sci Rep. 2021 Jul 8;11:14139. doi: 10.1038/s41598-021-93489-x (PMC8266831; doi:10.1038/s41598-021-93489-x)
Supplement: Supplementary file 1 — Supplementary Legends. [file 41598_2021_93489_MOESM1_ESM.docx]

**Dysregulation of anti-Mullerian hormone expression levels in mural granulosa cells of FMR1 premutation carriers**

**Running title:** **AMH dysregulation in FMR1 premutation carriers**

Moran Friedman-Gohas, MSc,^1^, Raoul Orvieto, MD,^1,2,3^, Abigael Michaeli, BSc,^1^, Adva Aizer, PhD,^2^, Michal Kirshenbaum, MD,^1,2^, Yoram Cohen, MD^1,2^

^1^Sackler Faculty of Medicine, Tel Aviv University, Tel Aviv, Israel; ^2^Infertility & IVF Unit, Chaim Sheba Medical Center, Tel Hashomer, Ramat Gan, Israel; ^3^The Tarnesby-Tarnowski Chair for Family Planning and Fertility Regulation at the Sackler Faculty of Medicine, Tel Aviv University, Tel Aviv, Israel

**Corresponding author and lead contact:** Yoram Cohen, MD, Infertility & IVF Unit, Chaim Sheba Medical Center, Tel Hashomer, Ramat Gan,5262000 Israel

Tel: +972-3-5302882, email: [ycohen1@gmail.com](mailto:ycohen1@gmail.com)

ORCID: http:/orcid.org/0000-0001-7242-0061

**Legend for supplementary figure 1 and supplementary table 1**

**Supplementary Figure 1. Similar SAM68 expression pattern in MCGs of *FMR1* premutation carriers and MGCs of a noncarrier.**

Fixated and stained MGCs from *FMR1* premutation carriers (*FMR* PM #2,4) displaying similar SAM68 expression compared to noncarrier (CTL #3). Bar = 20µm.

# Supplementary Table 1: Characteristics of FMRI premutation carriers
