# Supplementary figures and images for: Dysregulation of anti-Mullerian hormone expression levels in mural granulosa cells of FMR1 premutation carriers
Source: Sci Rep. 2021 Jul 8;11:14139. doi: 10.1038/s41598-021-93489-x (PMC8266831; doi:10.1038/s41598-021-93489-x)

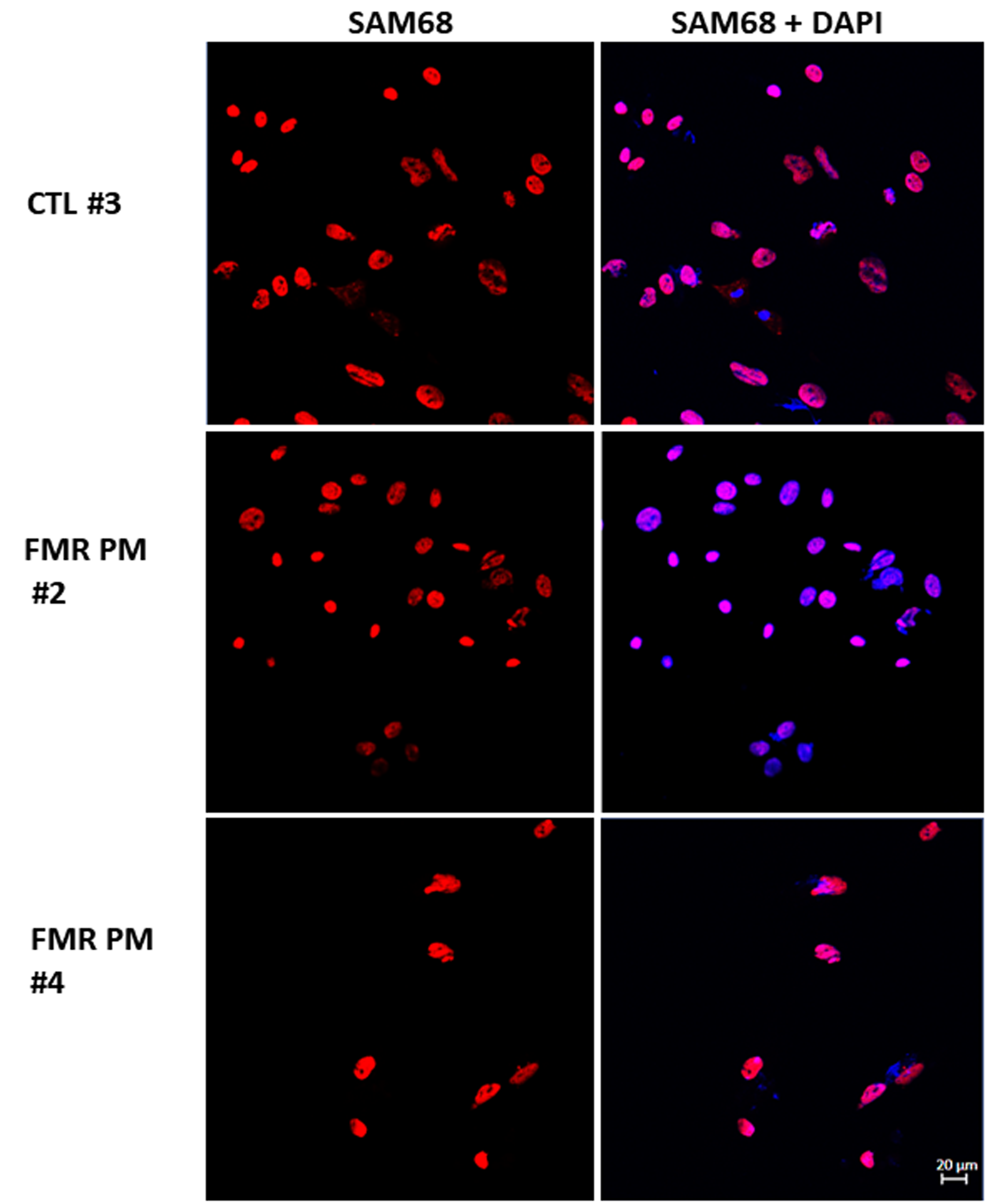

Supplement: Supplementary file 2 — Supplementary Figure. [file 41598_2021_93489_MOESM2_ESM.tif]
