# Supplementary material for: Dysregulation of anti-Mullerian hormone expression levels in mural granulosa cells of FMR1 premutation carriers
Source: Sci Rep. 2021 Jul 8;11:14139. doi: 10.1038/s41598-021-93489-x (PMC8266831; doi:10.1038/s41598-021-93489-x)
Supplement: Supplementary file 3 — Supplementary Table. [file 41598_2021_93489_MOESM3_ESM.docx]

**Supplementary Table 1 for:**

**Dysregulation of anti-Mullerian hormone expression levels in mural granulosa cells of *FMR1* premutation carriers**

**Running title:** **AMH dysregulation in *FMR1* premutation carriers**

Moran Friedman-Gohas, MSc,^1^, Raoul Orvieto, MD,^1,2,3^, Abigael Michaeli, BSc,^1^, Adva Aizer, PhD,^2^, Michal Kirshenbaum, MD,^1,2^, Yoram Cohen, MD^1,2^

| **Gene expression base line levels**  **(Relative to control)** | | | **Day3**  **Top quality embryo** | **ICSI***** | **INS**** | **Oocytes Number** | **Total dose of LH (IU)** | **Total dose of FSH (IU)** | **Protocol**  **used**  ******** | **Total days of stimulation** | **FSH initial dose (IU)** | **Peak of Estradiol (pmole/L)** | **Progesterone (nmole/L)** | **Basal LH (IU/L)** | **Basal FSH (IU/L)** | **BMI**  ***** | **Age (yrs)** | **CGG repeats number** |  |
| --- | --- | --- | --- | --- | --- | --- | --- | --- | --- | --- | --- | --- | --- | --- | --- | --- | --- | --- | --- |
| **FMR1** | **FSH receptor** | **AMH** | **Good responders** | | | | | | | | | | | | | | | | |
| 2.37 | NA | 6.73 | 5 | 6 | 5 | 11 | 900 | 1950 | 1 | 8 | 300 | 4633 | 1.3 | 4.7 | 8.1 | NA | 37.4 | 80 | 1 |
| NA | 1.52 | 0.43 | 4 | 15 | 5 | 20 | 787.5 | 2362.5 | 3 | 9 | 262.5 | 3894 | 5.2 | 5.4 | 6.9 | 21.5 | 27.8 | 64 | 2 |
| 0.15 | 8.82 | 2.88 | 1 | 9 | 9 | 18 | 750 | 2700 | 1 | 9 | 300 | 2254 | 2 | 5 | 5 | 27.6 | 33.7 | 199 | 3 |
| 0.75 | 2.16 | 0.23 | NA | NA | NA | 9 | 6000 | 6000 | 1 | 10 | 600 | 4831 | 2.1 | 3.9 | 3.4 | 20.2 | 37.2 | 82 | 4 |
| 1.39 | 5.77 | NA | 6 | 8 | 2 | 10 | 4800 | 4800 | 1 | 8 | 600 | 2750 | 1.6 | 2.7 | 7.2 | 25.8 | 26.9 | 123 | 5 |
| 1.32 | 4.78 | 0.57 | 9 | 20 | 0 | 20 | 0 | 1875 | 1 | 10 | 200 | 9266 | 2.1 | 5.9 | 5.1 | NA | 25.2 | 75 | 6 |
| 1.18 | 5.67 | 1.41 | 3 | 6 | 0 | 6 | 2250 | 3900 | 3 | 9 | 600 | 2738 | 1.4 | 5.5 | 4 | 22.7 | 30.6 | 83 | 7 |
| **Poor responders** | | | | | | | | | | | | | | | | | | | |
| 1.12 | 2.81 | 4.88 | 4 | 5 | 0 | 5 | 1950 | 5400 | 2 | 13 | 450 | 6476 | 7.8 | 3.7 | 5.2 | NA | 30.6 | 82 | 1 |
| 1.46 | 3.20 | 2.73 | 1 | 3 | 0 | 3 | 2100 | 4200 | 4 | 7 | 600 | 5426 | 8.7 | 1.3 | 2.5 | NA | 37.7 | 95 | 2 |
| 1.44 | NA | 1.12 | 3 | 0 | 3 | 3 | 2286 | 3486 | 1 | 12 | 300 | 8028 | 10.7 | 1.6 | 6.6 | 19.6 | 36.4 | 62 | 3 |
| 0.77 | 2.37 | 2.88 | 2 | 0 | 4 | 4 | 487.5 | 1387.5 | 1 | 6 | 225 | 2283 | 7.4 | 1.3 | 3.3 | 25.4 | 36.5 | 120 | 4 |
| 1.06 | 8.92 | 8.22 | 2 | 0 | 5 | 5 | 5100 | 5100 | 1 | 9 | 600 | 11461 | 8.7 | 3.2 | 2.5 | NA | 33.7 | 95 | 5 |

# Supplementary Table 1: Characteristics of *FMR1* premutation carriers

*BMI- Body mass index

**INS- Insemination

***ICSI- Intracytoplasmic sperm injection

****Protocol used:

1-Antagonist, 2- Long, 3- Ultrashort antagonist, 4- short
